# A Cross-sectional Study Investigating the Oral Health Status of Adult and Elderly Swiss Community-Dwellers 

**DOI:** 10.3290/j.ohpd.c_1794

**Published:** 2025-01-23

**Authors:** Roberta Borg-Bartolo, Andrea Roccuzzo, Christian Tennert, Maria Prasinou, Maurus Jäggi, Pedro Molinero-Mourelle, Martin Schimmel, Michael M. Bornstein, Guglielmo Campus

**Affiliations:** a; b Roberta Borg-Bartolo Researcher, PhD Student, Department of Restorative, Pediatric and Preventive Dentistry, School of Dental Medicine, University of Bern, Bern, Switzerland; Graduate School for Health Sciences, University of Bern, Bern, Switzerland. Data collection, wrote the manuscript, performed statistical analysis, analysed the results, proofread the manuscript.; c Andrea Roccuzzo Senior Lecturer, Department of Periodontology, School of Dental Medicine, University of Bern, Bern, Switzerland; Unit for Practice-based Research, School of Dental Medicine, University of Bern, Bern, Switzerland. Data collection, analysed the results, contributed substantially to discussion, proofread the manuscript.; d Christian Tennert Professor, Department of Restorative, Pediatric and Preventive Dentistry, School of Dental Medicine, University of Bern, Bern, Switzerland. Conceptualization, contributed substantially to discussion, proofread the manuscript.; e Maria Prasinou Dentist, Department of Restorative, Pediatric and Preventive Dentistry, School of Dental Medicine, University of Bern, Bern, Switzerland. Data collection, proofread the manuscript.; f Maurus Jäggi Dentist, Department of Restorative, Pediatric and Preventive Dentistry, School of Dental Medicine, University of Bern, Bern, Switzerland. Data collection, proofread the manuscript.; g Pedro Molinero-Mourelle Department of Reconstructive Dentistry and Gerodontology, School of Dental Medicine, University of Bern, Bern, Switzerland; Department of Conservative Dentistry and Prosthodontics, Faculty of Odontology, Complutense University of Madrid, Madrid, Spain. Contributed substantially to discussion, analysed the results, proofread the manuscript.; h Martin Schimmel Professor, Department of Reconstructive Dentistry and Gerodontology, School of Dental Medicine, University of Bern, Bern, Switzerland Professor. Conceptualization, advisor, proofread the manuscript.; i Michael M. Bornstein Professor, Department of Oral Health and Medicine, University Center for Dental Medicine Basel, University of Basel, Basel, Switzerland. Advisor, contributed substantially to discussion, proofread the manuscript.; j Guglielmo Campus Professor, Department of Restorative, Pediatric and Preventive Dentistry, School of Dental Medicine, University of Bern, Bern, Switzerland; Department of Cariology, Saveetha Dental College and Hospitals, SIMATS, Chennai, Tamil Nadu, India. Conceptualisation, performed statistical analysis, contributed substantially to discussion, analysed the results, proofread the manuscript.

**Keywords:** epidemiology, dental caries, oral health status, periodontal disease, public health

## Abstract

**Purpose:**

To evaluate the oral health status of community-dwellers ≥ 45 years of age in the canton of Bern, Switzerland.

**Materials and Methods:**

Data were collected using a questionnaire (including sociodemographic factors, medical history, oral health behaviour) and a clinical examination comprising caries, periodontal disease, oral hygiene, and prosthetic rehabilitation. χ^2^/Fisher’s tests and Cochrane Armitage trend tests as well as a binary logistic regression were performed to assess the association between oral disease presence (i.e., periodontal disease [PSI (periodontal screening index) score 3-4] and/or active dental caries [ICDAS 4-6, root ICDAS 2]) and the independent variables.

**Results:**

A total of 275 participants were included in the present study: 154 (56%) males and 121 (44%) females, with a mean age of 69.7 years (SD 11.6). The majority presented with good oral health behaviour; 221 (86%) brushed their teeth at least twice daily, 196 (79%) had regular dental visits. Nevertheless, 82 (32%) participants presented with an approximal plaque index of > 50%. The older age groups and participants with bleeding gums had higher odds of having active dental caries and/or periodontal disease (65-74 years – OR 2.88 [95% CI 1.33–6.25], ≥75 years – OR 2.60 [95% CI 1.17–5.78], bleeding gums OR 3.52 [95% CI 1.07–11.50]).

**Conclusion:**

The present study shows an association between age, oral hygiene, and the presence of active caries and periodontal disease. The study highlights the importance of good oral hygiene maintenance, especially in older adults.

As a result of high life expectancy rates and low birth rates, most countries worldwide have been experiencing an increase in elderly populations. The population of persons aged 65 years and over is 703 million worldwide, with this number expected to double in thirty years, while the number of persons aged 80 years and over is expected to triple by 2050.^39^ In Switzerland, there are currently over 1.5 million persons aged 65 years and over, and more than 2.4 million who fall within the 45-64-year age range (Swiss Federal Statistics Office, 2022).

Oral diseases, the most prevalent being periodontal disease and caries, are the most common non-communicable diseases worldwide,^17^ exceeding by far the number of cases of all other five main non-communicable diseases (i.e., mental disorders, cardiovascular disease, diabetes mellitus, chronic respiratory diseases, and cancers) combined.^42^ Several risk factors have been associated with a higher prevalence of oral disease in the elderly, including frailty,^11,20^ care-dependency and cognitive conditions,^8,10^ multi-morbidity and polypharmacy,^9^ as well as a reduction in the utilisation of dental services.^14^ A consequence of untreated oral diseases is tooth loss – the greatest impact on quality of life from a patient’s perspective.^3,12^ Oral diseases and poor oral health impact an individual’s quality of life^40^ and influence both food intake^24^ as well as social interactions.^4^ Thus, preventing the development and progression of oral diseases and maintaining good oral health is essential throughout the life course of an individual.

To date, very few studies have assessed the oral health status of non-institutionalised adult and elderly persons in Switzerland.^1,33^ The study aims to reduce the currently existing gap, by describing and analysing the oral health status of non-institutionalised persons of at least 45 years of age in the canton of Bern. The null hypothesis for this study was that there was no association between age and oral health status.

## MATERIALS AND METHODS

This study is designed as a cross-sectional, mono-centric study with participants aged ≥ 45 years, living in the community in the canton of Bern, Switzerland. The study was conducted according to the revised principles of the Helsinki Declaration (2013). Before the start of the study, ethical approval was obtained from the Ethics Committee of the Canton of Bern (KEK), Switzerland (Nr. 2020-02760, Nr. 2021-01947). Data reporting follows the STROBE guidelines.

The methodology of the study has been described in a previous publication.^33^ Briefly, the participants were chosen by random sampling from the ten regions of the canton of Bern by proportional allocation, and for each region, the “probability proportional to size” sampling method was applied.^41^ The participants were first contacted by mail and detailed study information was provided. Once participants agreed to take part in the study and gave their written informed consent, they were asked to fill out a questionnaire with questions on sociodemographic factors, medical history, oral health behaviour, and nutrition. Finally, a clinical examination was performed at the participant’s place of residence. Data collection was carried out between January 2022 and December 2023.

### Clinical Examination 

The examination was carried out using a planar mirror (Hahnenkratt; Königsbach-Stein, Germany), a WHO ball-ended probe (Asa-Dental, Milan, Italy), and a headlamp as a light source. Two experienced dentists (AR, RBB), two master dental students (MP, MJ), and four undergraduate dental students carried out data collection.

### Clinical Assessment

The following data were collected during the clinical examination:

Oral hygiene: Approximal Plaque Index (API)^23^ and modified Papilla Bleeding Index (mPBI)^29^ – the mesial and distal interproximal spaces of the second and fourth quadrants (buccal surface) and the first and third quadrants (palatal/lingual surface) were examined using a periodontal probe, and presence or absence of dental plaque (API) or bleeding gums (m PBI) was recorded.
Periodontal disease: Periodontal Screening Index (PSI): each tooth present was assessed at six sites and the highest score per sextant was recorded. The scores range from 0 (healthy periodontal tissue) to 4 (probing pocket depth > 5 mm).
Caries: The International Caries Detection and Assessment System (ICDAS) was used to record the presence of caries (coronal and root) per tooth present. Initial caries was recorded as ICDAS 1-3 or root ICDAS 1, active caries as ICDAS 4–6 or root ICDAS 2.
Prosthetic rehabilitation: removable dental prostheses (full or partial dentures), fixed dental prostheses (crowns, bridges, implants)
Missing and filled teeth: the DMFT (decayed, missing, filled teeth) index was calculated.

### Questionnaire

The questionnaire collected data on the following variables: age: categorised into three age groups (45–64, 65–74, ≥ 75 years); gender: male, female; location: urban, rural (to be considered urban, the cut-off was taken to be at least 10,000 inhabitants for a given area^36^); employment status: employed, retired; educational level: having tertiary education, not having tertiary education; marital status: married, not married; medical history: cardiovascular disease, gastrointestinal problems, cancer, thyroid disease, diabetes, rheumatoid arthritis, depression, smoking, regular consumption of alcohol; oral health habits: toothbrushing frequency: at least twice daily, less than twice daily; use of dental adjuncts: mouthwash yes/no, dental floss and/or interdental brushes yes/no; frequency of visits to the dentist/dental hygienist: within the last 12 months/over 12 months; sugar consumption: yes/no.

The study data collected were anonymously stored and managed using a dedicated software program (REDCap; Vanderbilt University, Nashville, TN, USA).

### Calibration

Before the commencement of the study, the examiners were trained and calibrated on the classification of carious lesions and dental restorations using the ICDAS, with details on the methodology having been published previously for the pilot study carried out before the current study.^33^ Intra-rater reliability and inter-rater reliability were calculated using Cohen’s kappa scores and intra-class correlation coefficients (ICC) using the two-way mixed effects model, respectively. Kappa scores of 80–100% (p < 0.05, caries lesion calibration) and 100% (p < 0.05, dental restoration calibration) were obtained. The average ICCs for inter-rater reliability were 0.97 (95%CI = 0.94 – 0.98, p < 0.05) (caries, first calibration session), 0.95 (95%CI = 0.90–9.98, p < 0.05) (caries, second calibration), and 0.98 (95%CI = 0.97–0.99, p < 0.05) (dental restoration, first and second calibration).

### Sample Size Calculation

Sample size calculation was performed before the start of the pilot study.^33^ As the prevalence of active oral diseases (periodontal disease and caries) in the pilot study were found to be lower than assumed, a post-hoc power analysis was performed. An assumed prevalence of active oral diseases of 50% ^33^ and a prevalence of 9% active caries and 41% active periodontal disease in the present study, a sample size of 275 participants, and a standard error of 0.05, a 0.85 power was achieved.

### Statistical Analysis

Statistical analysis was carried out using Stata SE18® (Stata; College Station, TX, USA) with statistical significance set at p < 0.05. Descriptive statistics were performed with means and standard deviation (SD) to describe continuous variables and the number of participants (n) and frequency (%) for categorical variables. A binary variable was created for participants with at least one oral disease, i.e., the presence of periodontal disease (PSI score 3–4) and/or the presence of active caries (ICDAS 4–6, root ICDAS 2). Chi-squared tests, Fisher’s tests, and Cochrane Armitage trend tests were performed to assess the crude association between the dependent binary variable (presence of periodontal disease or caries) and the independent variables. Unadjusted and adjusted models were used to study the association between the dependent and independent variables. Five binary logistic regression models were carried out; Model 1 included only age. Demographic factors were added in Model 2, with medical conditions added in Model 3. Model 4 consisted of oral health behaviour factors, and oral hygiene factors were added in Model 5.

## RESULTS

**Fig 1:**
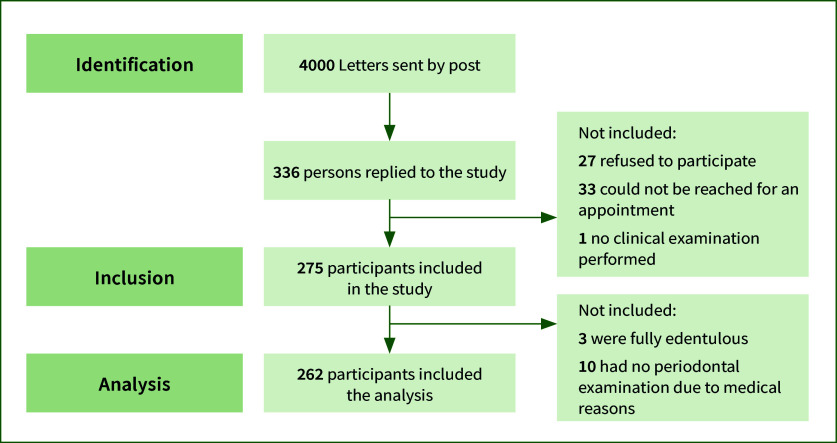
STROBE flow diagram.

Out of the 4000 letters initially sent out, 336 persons responded (8.4% response rate), out of which 33 could not be reached for an appointment and 27 refused to participate. Thus, 275 participants were included in the study, filled out the questionnaire, and underwent a clinical examination (Fig 1). There were 154 (56%) males and 121 (44%) females with a mean age of 69.7 years (SD 11.6) and a range of 45–99 years. Of the study participants, 90 (73%) lived in rural areas, 167 (66%) were married and 139 (53%) had a tertiary level of education. The most prevalent medical condition was high blood pressure (33%), followed by cardiovascular disease (15%) and rheumatoid arthritis (12%). The majority brushed their teeth at least twice daily (221/86%) and visited the dentist regularly (n = 196, 79%). Almost a third of the participants (82/32%) presented with an API > 50%, while 22 (8%) had a mPBI >50% (Table 1).

**Table 1:** Participants’ characteristics classified by presence or absence of active periodontal disease/caries

		123 (46.9)	139 (53.1)		p-value	z-value	exact probability
Sex	Female	48 (41.0)	69 (59.0)	117 (44.7)			
	Male	75 (51.7)	70 (48.3)	145 (55.3)			
				262 (100)	0.09	1.72	0.11
Age	45-64 years	22 (28.6)	55 (71.4)	77 (29.4)			
	65-74 years	51 (54.3)	43 (45.7)	94 (35.9)			
	≥75 years	50 (49.5)	41 (45.1)	91 (34.7)			
				262 (100)	**<0.01**	3.31	**<0.01**
Location	Urban	35 (48.6 )	37 (51.4)	72 (27.5)			
	Rural	88 (46.3)	102 (53.7)	190 (72.5)			
				262 (100)	0.74	0.33	0.83
Marital status	Married	84 (50.3)	83 (49.7)	167 (65.5)			
	Not married	39 (43.3)	51 (56.7)	90 (34.4)			
				257 (100)	0.33	0.98	0.39
Educational level	Tertiary	67 (48.2)	72 (51.8)	139 (53.1)			
	No tertiary education	56 (45.5)	67 (54.5)	123 (46.9)			
				262 (100)	0.66	0.43	0.78
Employment status	In employment	34 (34.7)	64 (65.3)	98 (37.4)			
	retired	89 (54.2)	75 (45.7)	164 (62.6)			
				262 (100)	**<0.01**	-3.07	**<0.01**
High blood pressure	No	72 (40.9)	104 (59.1)	176 (67.2)			
	Yes	51 (59.3)	35 (40.7)	86 (32.8)			
				262 (100)	**<0.01**	2.8	**<0.01**
Cardiovascular disease	No	105 (47.5)	116 (52.5)	221 (84.4)			
	Yes	18 (43.9)	23 (56.1)	41 (15.36)			
				262 (100)	0.67	-0.42	0.67
Rheumatoid arthritis	No	107 (46.5)	123 (53.5)	230 (87.8)			
	Yes	16 (50.0)	18 (50.0)	32 (12.2)			
				275 (100)	0.71	0.36	0.71
Depression	No	110 (46.6)	126 (53.4)	236 (90.1)			
	Yes	13 (50.0)	13 (50.0)	26 (10.0)			
				262 (100)	0.74	0.32	0.74
Diabetes	No	105 (44.3)	132 (55.7)	237 (90.5)			
	Yes	18 (28.0)	7 (72.0)	25 (9.5)			
				262 (100)	**0.01**	2.63	**<0.01**
Toothbrushing frequency	Twice daily or more	101 (45.7)	120 (54.3)	221 (85.7)			
	Once daily or less	16 (48.5)	17 (51.5)	33 (14.3)			
				254 (100)	0.85	-0.3	0.76
Type of toothbrush	Electric	63 (53.3)	55 (46.6)	118 (45.6)			
	Nanual	57 (40.4)	84 (59.6)	141 (54.4)			
				259 (100)	**0.04**	2.08	**0.04**
Use of fluoride toothpaste	Yes	106 (48.6)	112 (51.4)	218 (85.8)			
	No	12 (33.3)	24 (66.7)	36 (14.2)			
				254 (100)	0.11	1.7	0.08
Use of mouthwash	Yes	78 (50.3)	77 (49.7)	155 (59.8)			
	No	42 (40.3)	62 (59.6)	104 (40.2)			
				259 (100)	0.12	1.57	0.12
Use of interdental brushes/floss	Yes	95 (45.9)	112 (54.1)	207 (88.1)			
	No	16 (57.1)	12 (42.9)	28 (11.9)			
				235 (100)	0.26	-1.11	0.26
Visit to the dentist	In the last 12 months	72 (36.7)	124 (63.3)	196 (79.4)			
	Over 12 months ago	21 (41.2)	30 (58.9)	51 (20.6)			
				247 (100)	0.56	0.62	0.53
Visit to dental hygienist	In the last 12 months	90 (46.8)	102 (53.1)	192 (80.6)			
	Over 12 months ago	20 (47.6)	22 (52.3)	42 (19.4)			
				234 (100)	0.93	0.09	0.93
Sugar intake	Not daily	18 (40.9)	26 (59.1)	44 (16.9)			
	At least once daily	104 (48.2)	112 (51.9)	216 (83.1)			
				260 (100)	0.38	0.87	0.38
Approximal Plaque Index (API)	0-49%	76 (42.7)	102 (57.3)	178 (68.5)			
	50-100%	47 (57.3)	35 (42.6)	82 (31.5)			
				260 (100)	**0.03**	2.19	**0.03**
Modified Papilla Bleeding Index (mPBI)	0-49%	106 (44.5)	132 (55.4)	238 (91.5)			
	50-100%	17 (77.2)	5 (22.7)	22 (8.5)			
				260 (100)	**<0.01**	2.93	**<0.01**
Missing teeth	0 missing teeth	21 (31.3)	46 (68.7)	67 (25.5)			
	<10 missing teeth	93 (52.2)	85 (47.7)	178 (67.9)			
	>10 missing teeth	9 (52.9)	8 (47.1)	17 (6.5)			
				262 (100)	**0.01**	2.65	**<0.01**
Filled teeth	0-4 filled teeth	22 (34.3)	42 (65.6)	64 (24.6)			
	5-10 filled teeth	40 (42.1)	55 (57.8)	95 (36.5)			
	>10 filled teeth	61 (60.4)	40 (39.6)	101 (38.8)			
				260 (100)	**<0.01**	3.4	**<0.01**
Removable prosthesis	No	118 (47.7)	129 (52.2)	247 (94.3)			
	Yes	5 (33.3)	10 (66.6)	15 (5.7)			
				262 (100)	0.30	-1.08	0.27
Fixed prosthesis	No	37 (41.1)	53 (58.8)	90 (34.4)			
	Yes	86 (50.0)	86 (50.0)	172 (65.6)			
				262 (100)	0.19	1.36	0.21
Implants	No	108 (49.1)	122 (50.9)	220 (83.9)			
	Yes	15 (35.7)	27 (64.3)	42 (16.0)			
				262 (100)	0.11	-1.59	0.11

The mean number of decayed, missing, and filled teeth was 0.18 (SD 0.76, range 0-8 teeth), 3.76 (SD 5.21, range 0–28 teeth), and 8.76 (SD 5.17, range 0–21 teeth), respectively. The mean DMFT was 12.71 (SD 5.86, range 0–28 teeth). The mean number of teeth with root caries was 0.12 (SD 0.90, range 0–11 teeth). Of the included participants, 108 (41%) presented with periodontal disease (PSI scores 3–4), 25 (10%) had active caries (ICDAS 4–6, root ICDAS 2), 9 (3%) participants presented with root caries (root ICDAS 2), and 36 (13%) participants had initial carious lesions (ICDAS 1–3) (Table 2).

**Table 2:** Prevalence of periodontal disease and active caries by age group

45-64	20 (18.5)	57 (37.0)	77 (29.3)	5 (20.0)	73 (29.2)	78 (28.4)
65-75	42 (38.9)	52 (33.7)	94 (35.8)	12 (48.0)	89 (35.6)	101 (36.7)
≥75	46 (42.6)	45 (29.2)	91 (34.7)	8 (32.0)	88 (35.2)	96 (34.9)
	108 (41.2)	154 (58.7)	262 (100)	25 (9.01)	250 (90.9)	275 (100)


Very few participants were fully edentulous (3/1%), with one participant aged 65–74 years and the other two participants aged over 75 years. The number of participants who had at least one tooth crowned was 189 (69%), 15 (5%) had a removable prosthesis, 172 (63%) had at least one fixed prosthesis, and 42 (15%) were rehabilitated with implants. In the final statistical analysis, 262 participants were included, as 13 participants could not undergo a periodontal examination (3 fully edentulous, 10 due to medical conditions). Almost half of the study participants (123/47%) had either active periodontal disease or active dental caries or both.

**Fig 2:**
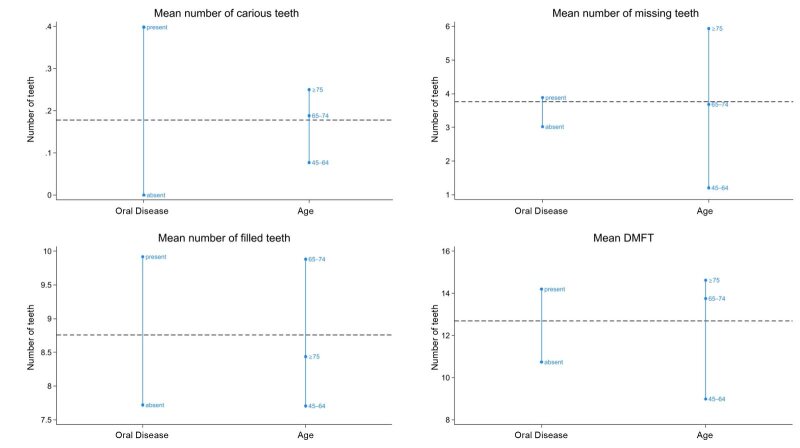
Mean number of decayed, missing and filled teeth, mean DMFT by age group and presence or absence of active periodontal disease/caries.

χ^2^ and/or Fisher’s exact tests showed a significant association between having periodontal disease and/or dental caries and age, presence of high blood pressure and diabetes, using an electric or manual toothbrush, poor oral hygiene (API, mPBI), and number of missing and filled teeth (Table 1). The participants with periodontal disease and/or dental caries had a higher mean number of missing teeth (3.88 teeth), filled teeth (9.91 teeth), and a higher DMFT (14.02) than the group without active periodontal disease/caries (3.02 missing teeth, 7.72 filled teeth, 10.74 DMFT). The younger age groups had fewer missing teeth, decayed teeth, and a lower DMFT, while the 65-year-old age group had the highest mean of filled teeth (Figure 2). The binary logistic regression showed that age remained statistically significant in all 5 models, with the older age groups having higher odds of having active periodontal disease and/ or dental caries (65–74 years – OR 2.88 (95% CI 1.33–6.25), ≥ 75 years – OR 2.60 (95% CI 1.17–5.78)) while participants with bleeding gums (mPBI) had 3.51 (95% CI 1.07–11.50) higher odds. Using dental floss was just on the 0.05 statistical significance cut-off, showing that participants who used dental floss regularly had lower odds of having active periodontal disease and/or caries (OR 0.42, 95% CI 0.15–1.21) (Table 3).

**Table 3:** Binary regression analyses – Models 1 to 5

Age	45–64 years	1	1	1	1	
	65–74 years	2.96 (1.56-5.62)	2.92 (1.51-5.63)	2.51 (1.27-4.95)	2.95 (1.38-6.32)	2.88 (1.33-6.25)
	≥75 years	3.05 (1.60–5.81)	3.23 (1.65–6.34)	2.90 (1.43–5.87)	2.63 (1.21–5.74)	2.60 (1.17–5.78)
Sex	Female		1	1	1	1
	Male		1.33 (0.79–2.25)	1.37 (0.80–2.34)	1.21 (0.66–2.23)	1.26 (0.68–2.34)
Marital status	Not married		1		1	1
	Married		1.33 (0.77–2.27)	1.44 (0.82–2.51)	1.72 (0.91–3.24)	1.93 (1.00–3.73)
High blood pressure	Not present			1	1	1
	Present			1.59 (0.89–2.84)	1.53 (0.80–2.96)	1.55 (0.79–3.03)
Diabetes	Not present			1	1	1
	Present			2.44 (0.92–6.48)	2.89 (0.96–8.75)	2.33 (0.74–7.37)
Cardiovascular disease	Not present			1	1	1
	Present			0.56 (0.26–1.19)	0.42 (0.18–0.99)	0.40 (0.16–0.97)
Toothbrushing	Manual toothbrush				1	1
	Electric toothbrush				1.75 (0.98–3.17)	1.79 (0.99–3.26)
Use of fluoride toothpaste	No				1	1
	Yes				1.95 (0.81–4.75)	1.63 (0.66–4.02)
Use of dental floss	No				1	1
	Yes				0.37 (0.14–0.99)	0.42 (0.15–1.21)
Frequency of dental visits	<12 months				1	1
	>12 months				1.62 (0.75–3.46)	1.60 (0.74–3.48)
Sugar	No				1	1
	Yes				1.63 (0.75–3.54)	1.64 (0.73–3.66)
Use of mouthwash	No				1	1
	Yes				1.69 (0.93–3.07)	1.69 (0.93–3.09)
Papilla Bleeding Index	No					1
	Yes					**3.51 (1.07–11.50)**

## DISCUSSION

The present study aimed to provide a comprehensive overview of the oral health conditions of Swiss community-dwellers aged ≥ 45 years. To the best of the authors’ knowledge, this is the first study that has been carried out in Switzerland, where adults and elderly persons living in the community, were recruited directly from municipalities by random sampling, and a comprehensive oral health assessment was performed, with data collected on the oral health status by a clinical examination, which included a periodontal and caries assessment. Thus, the study provides novel findings that fill a gap in the data currently existing in Switzerland. Based on the obtained results, it can be stated that despite good self-reported oral hygiene habits, high levels of plaque and bleeding scores were detected. Half of the participants had either active periodontal disease, active caries or both, while the older age groups presented with a poorer oral health status compared to the younger age groups, having more missing teeth and fewer filled teeth. Age and a high full-mouth bleeding score were associated with the presence of active periodontal disease and/or caries.

In the present study, the oldest age group (≥75 years), had almost twice the number of missing teeth compared to the study average. On the other hand, the 65–74-year group, had more teeth present and more filled teeth. These factors contribute to a higher DMFT in the participants aged 65 years and over. These are age-related effects that are likely attributed to the cumulative effect detectable in the elderly compared to younger individuals.^25^ When compared to the latest available published national data, dated from 2012, the study participants presented with a lower mean number of missing teeth.^36^ Direct comparison is difficult, as national data were collected only by self-reported means and the present study might not be representative of the national population. However, such differences could also reflect a trend that the number of missing teeth in the elderly population is decreasing.^21,27^

The prevalence of periodontal disease was higher in participants aged ≥ 65 years compared to the youngest age group, findings that are in line with similar studies.^35^ In this study population, the prevalence of active caries was lower compared to the prevalence reported in studies conducted in Germany (21%; 65–74 years)^19^ and England and Wales (40%; 75–84 years).^26^ Periodontal disease and caries are associated with several general health conditions, such as cardiovascular disease,^15^ diabetes,^28^ and rheumatoid arthritis.^13^ In Switzerland, about one-half of the persons aged 55 years and over, and two-thirds of persons aged 75 years and over, have at least one chronic disease,^18^ the most common being high blood pressure, cardiovascular disease, diabetes, and rheumatoid arthritis.^18^ Although a direct causality could neither be investigated nor detected, several risk factors for dental caries and periodontal disease have been identified, including polypharmacy,^37^ as well as impaired oral hygiene maintenance.^32^ In the current study, the presence of high blood pressure and diabetes were found to be statistically significantly associated with active periodontal disease and/or caries, though losing their significance in the adjusted regression model. Despite these results, the bidirectional link between general health status and oral health status is not to be underestimated,^34^ and maintaining good oral health is an integral part of general health and well-being.^43^

A high mPBI was found to be statistically significantly associated with the presence active periodontal disease and/or caries. An interesting finding in the current study is that, although the majority of the participants reported good oral hygiene habits, one-third of the participants had plaque present at least on one-half of the examined interproximal spaces, with one in ten participants experiencing bleeding on probing. Although the participants had good oral health awareness and practiced good oral hygiene habits, their oral hygiene status did not reflect their self-perceived oral health care behaviour. One potential explanation of this finding is the discrepancy between a person’s self-reported frequency of at-home oral hygiene measures and the detected plaque values, reflecting the challenges in performing adequate daily plaque control, which is even more challenging in elderly populations.^30,38^

As people age, their ability to care for themselves decreases. The overall oral health status of the study participants was better compared to similar studies conducted in Switzerland on care-dependent community dwellers^1,22^ and nursing home residents.^2,5,6^ Although the care-dependency status was not recorded in the present study, it is assumed that the participants had a low level of care-dependency, or none at all, while the mean age was younger than in the cited studies.^1,5,6,22^ Declines in oral health status with the onset of care dependency have been widely documented. In dental practices, dentists need to implement dedicated interventional measures to improve patients’ awareness of oral health issues associated with aging, such as the effects that systemic conditions and medications have on oral health.^16,31^

Methodologically, the study has several strengths; the data collection methods included both questionnaires and clinical examination, which enabled data collection on oral health factors such as oral hygiene, periodontal disease, and caries. The random sampling of the participants and the good representation of all the ten regions of the canton of Bern are to be counted as strong points of the study. Limitations include the participants’ low response rate. However, the response rate is comparable to similar studies conducted in Switzerland.^1^ The cross-sectional design of the study is a further limitation as it does not allow for causal interpretation of the results while a potential participation bias cannot be excluded since the majority of the participants were highly educated, leading to people from lower education backgrounds being under-represented.

In light of the current findings, clinical implications include the need to instruct patients in the dental practice on proper oral hygiene maintenance, taking into consideration the needs of the patient on an individual level, e.g., demonstrating and practicing the right technique for cleaning in the presence of dental prostheses or modified cleaning methods for participants with impaired manual dexterity.^32^ On a national and international level, the current research findings highlight the need for standardised nationwide data collection and assessment in countries worldwide, to enable the planning and effective evaluation of need-based oral health policies and services. Across countries, such data would allow for comparisons of the impact of different oral health policies and benchmarking of oral health and services.^7^

## CONCLUSION

The present study, within its limitations, shows an association between age, oral hygiene, and the presence of active dental caries and periodontal disease. It highlights the importance of good oral hygiene maintenance, especially in older adults, to ensure a good oral health status at all ages.
